# The Cyclical Development of *Trypanosoma vivax* in the Tsetse Fly Involves an Asymmetric Division

**DOI:** 10.3389/fcimb.2016.00115

**Published:** 2016-09-28

**Authors:** Cher-Pheng Ooi, Sarah Schuster, Christelle Cren-Travaillé, Eloise Bertiaux, Alain Cosson, Sophie Goyard, Sylvie Perrot, Brice Rotureau

**Affiliations:** ^1^Trypanosome Transmission Group, Trypanosome Cell Biology Unit, Department of Parasites and Insect Vectors, Institut Pasteur and INSERM U1201Paris, France; ^2^Trypanosomatids Infectious Processes Unit, Department of Infection and Epidemiology, Institut PasteurParis, France

**Keywords:** *Trypanosoma vivax*, tsetse fly, parasite cycle, differentiation, asymmetric division, development

## Abstract

*Trypanosoma vivax* is the most prevalent trypanosome species in African cattle. It is thought to be transmitted by tsetse flies after cyclical development restricted to the vector mouthparts. Here, we investigated the kinetics of *T. vivax* development in *Glossina morsitans morsitans* by serial dissections over 1 week to reveal differentiation and proliferation stages. After 3 days, stable numbers of attached epimastigotes were seen proliferating by symmetric division in the cibarium and proboscis, consistent with colonization and maintenance of a parasite population for the remaining lifespan of the tsetse fly. Strikingly, some asymmetrically dividing cells were also observed in proportions compatible with a continuous production of pre- metacyclic trypomastigotes. The involvement of this asymmetric division in *T. vivax* metacyclogenesis is discussed and compared to other trypanosomatids.

## Introduction

African trypanosomiases are a set of vector-borne diseases of humans and their livestock resulting from infections with flagellated unicellular parasites named African trypanosomes (Kinetoplastida: Trypanosomatidae) that are almost exclusively transmitted by the bite of tsetse flies (Diptera: Glossinidae). At least seven trypanosome species cause Animal African Trypanosomiasis (AAT) (review in Rotureau and Van Den Abbeele, 2013). Among these, *Trypanosoma (Duttonella) vivax, T. (Nannomonas) congolense*, and to a lesser extent *T. (Trypanozoon) brucei brucei*, are the major pathogens of cattle and other ruminants. AAT threatens about 50 million heads of cattle and causes about 3 million deaths annually. It has a marked impact on agriculture in sub-Saharan endemic countries, leading to annual livestock production losses of about 1.2 billion US dollars (FAO, 2015). AAT restricts agricultural development on the African continent despite the availability of prophylactic and curative drugs. Moreover, drug effectiveness is being seriously threatened by increasing drug resistance in animal trypanosomes (Delespaux et al., 2008).

*T. vivax* is a major pathogenic trypanosome of domestic animals and is the dominant species in West Africa both in terms of geographic distribution and prevalence (Gardiner and Wilson, 1987; Osorio et al., 2008; FAO, 2015). Infections in cattle, also termed *nagana, souma*, or *gobiat*, are accompanied by weight loss, reduced milk yields, stillbirths, abortions, and mortality. Sheep, goats, horses, and camels also suffer pathogenic effects following *T. vivax* infection. *T. vivax* is predominantly transmitted by tsetse flies following cyclical development. Although mechanical transmission by biting flies other than tsetse flies such as horse flies (tabanids) and stable flies (*Stomoxys*) does occur in Africa, its relative importance to the epidemiological picture has been questioned. *T. vivax* can be cyclically transmitted by at least nine species of tsetse (especially *G. morsitans* spp., *G. longipalpis, G. palpalis, G. tachinoides*, and *G. pallidipes*) in which the development of infective metacyclic trypanosomes can take 3–13 days, depending on the parasite strain, the vector competence, and the thermo-hygrometric parameters of the environment (Gardiner and Wilson, 1987; Osorio et al., 2008; FAO, 2015).

A tsetse fly ingests trypanosomes during the acquisition of a blood meal on an infected mammal. Within the fly, the parasites have to go through a precise developmental cycle that culminates in the differentiation into infective metacyclic trypanosomes that are ready for transmission to the next mammalian host.

Trypanosomes possess a nucleus (N) and a single mitochondrion whose genetic material is condensed in a structure termed the kinetoplast (K) that is linked to the basal body apparatus of the flagellum (Hoare, 1966; Robinson and Gull, 1991). The flagellum is attached to the cell body and tracts the trypanosome forward, hence defining the antero-posterior axis of the cell. Two main characteristic morphotypes have been defined according to the relative position of the kinetoplast to the nucleus (Hoare, 1966). In trypomastigotes (T or Trypo), the kinetoplast localizes between the nucleus and the posterior end of the cell, whereas in epimastigote forms (E or Epi) it is positioned between the nucleus and the anterior end of the cell. A remarkable common feature in the parasite cycle progression of salivarian trypanosomes is the switch between these two morphotypes (Hoare, 1972; Rotureau and Van Den Abbeele, 2013).

**Figure 1:**
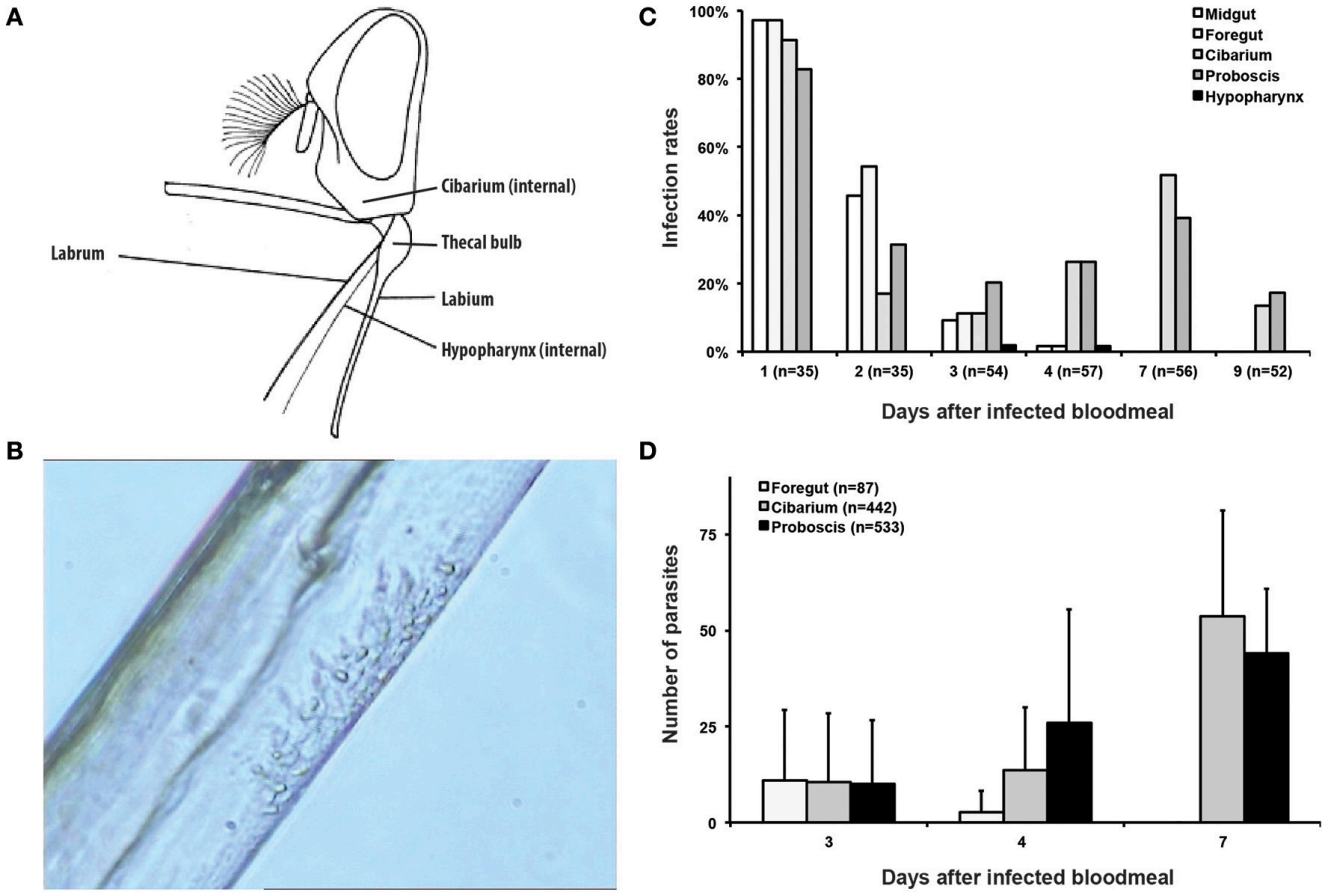
*****T. vivax*** distribution, infection rates and parasite densities during cyclical development in the tsetse fly. (A)** Illustration of the head of *Glossina* in profile with the mouthparts spread out artificially [modified from FAO (1992)] showing the anatomy of the mouthparts where *T. vivax* development occurs. **(B)** Still image from movie S1 showing an islet of *T. vivax* epimastigotes attached to the chitinous lining of the labrum in the median region of a proboscis. **(C)** Histogram of the infection rates (in % of dissected flies) in different regions of the mouthparts according to the days after ingestion of the infected bloodmeal. The total number of flies dissected each day is indicated in brackets. **(D)** Histogram of the parasite densities in different regions of the mouthparts (total number of parasites/organ/fly) according to the days after ingestion of the infected bloodmeal. The total number of parasites per organ, including both attached and flushed trypanosomes, is indicated in the legend.

Although *T. vivax* development appears to be simpler than that of *T. b. brucei* and *T. congolense*, it remains poorly studied (Rotureau and Van Den Abbeele, 2013). Most of the few observations, including the key description of some developmental stages in the sub-genus *Duttonella*, were published more than 75 years ago by Bruce et al. (1910, 1911), Lloyd and Johnson (Lloyd and Johnson, 1924), Roubaud (Roubaud, 1935), and reviewed by Hoare (1972). The classical theory was that cyclical development of *T. vivax* in *Glossina* was entirely confined to the proboscis, i.e., the labium, labrum, and hypopharynx (Figure 1A, Lloyd and Johnson, 1924; Roubaud, 1935). However, *T. vivax* parasites were also observed in the cibarium (pharynx) and proboscis of *G. palpalis* with mature parasite infections by Bruce et al. (1911). More recently, a similar observation was made in *G. tachinoides* (Jefferies et al., 1987) and in *G. morsitans* (Moloo and Gray, 1989). Therefore, it is likely that, at least in a number of tsetse, *T. vivax* cyclical development initially occurs in the cibarium/oesophageal region from where parasites migrate to the proboscis to complete their development.

Nevertheless, the available overall picture does not explain the continuous production of infective metacyclic parasites throughout the life of the vector. The exact developmental progression from the trypomastigote to the epimastigote, and back to the trypomastigote morphotype remains to be unraveled: does it involve asymmetric divisions as described in other trypanosomes (Van Den Abbeele et al., 1999; Sharma et al., 2008; Rotureau et al., 2012; Kurup and Tarleton, 2014) or does it depend on differentiation events in non-dividing cells (Rotureau et al., 2011; Rico et al., 2013)?

*T. vivax* infections in tsetse flies are characterized by: (i) high infection rates observed in various species (from 10 to 80%), (ii) an irregular frequency of metacyclic parasite release in the saliva, and (iii) some very low numbers of metacyclic forms extruded in these rare cases (approximately 1–20 parasites depending upon the strain, i.e., 50–100-fold less than for *T. brucei*) (Bruce et al., 1910, 1911; Lloyd and Johnson, 1924; Roubaud, 1935; Otieno and Darji, 1979). These characteristics have probably reduced or even precluded direct examination of metacyclogenesis in the past. Our knowledge on *T. vivax* development is currently very limited, but the increasing socio-economic impact of this widespread parasite prompts in-depth investigation of its life cycle. We therefore scrutinized how trypanosomes proliferate in the cibarium and proboscis, and how these processes could be coupled to differentiation.

## Materials and methods

### *T. vivax* strain, maintenance and culture

*Trypanosoma (Dutonella) vivax* IL 1392 was originally derived from the Zaria Y486 Nigerian isolate (Chamond et al., 2010; Goyard et al., 2014). These parasites had been characterized and were maintained in the laboratory by continuous passage in mice, as previously described (Chamond et al., 2010; Goyard et al., 2014). 10-week-old male Swiss Outbred (CD-1, RJOrl:SWISS) were used in all experiments. Mice were intra-peritoneally (i.p.) injected with bloodstream forms of *T. vivax* and parasitaemia was determined as previously described (Chamond et al., 2010). In order to reduce the number of animals, all the infected mice were re-used from other studies (D'Archivio et al., 2011, 2013; Goyard et al., 2014 and unpublished data) in which they would have otherwise been directly sacrificed after parasite passages for maintenance. All animal work was conducted in accordance with relevant national and international guidelines (see below). Alternatively, epimastigote *T. vivax* cells were cultured and differentiated in axenic conditions as previously described (D'Archivio et al., 2011).

### Ethical statements

This study was carried out in strict accordance with the recommendations in the Guide for the Care and Use of Laboratory Animals of the European Union (European Directive 2010/63/UE) and the French Government. Experimental infection procedures were approved by the *Comité d'Ethique Paris Centre et Sud* #59 (Permit #2012-0043). Animal housing conditions and the protocols used in the work described herein were approved by the “*Direction des Transports et de la Protection du Public, Sous-Direction de la Protection Sanitaire et de l'Environnement, Police Sanitaire des Animaux”* under number B-75-15-28, in accordance with the Ethics Charter of animal experimentation that includes appropriate procedures to minimize pain and animal suffering. BR is authorized to perform experiments on vertebrate animals (license #A-75-2035) and is responsible for all the experiments conducted personally or under his supervision.

### Tsetse fly maintenance, infection and dissection

Teneral males of *Glossina morsitans morsitans* from 8 to 96 h post-eclosion were allowed to feed on mice infected with *T. vivax* at a parasitaemia comprised between 10^6^ and 10^8^ parasites/ml. Mice were first anesthetized by i.p. injection of 100 μl of a mix solution of Ketamine (Imalgene1000 at 125 mg per kg bodyweight) and Xylazine (Rompun 2% at 12.5 mg per kg bodyweight) and individually laid for 20 min on the upper net of a Roubaud cage containing no more than 50 tsetse flies. Mice were subsequently sacrificed by cervical dislocation before waking-up. Tsetse flies were subsequently maintained in Roubaud cages up to 15 days at 27°C and 70% hygrometry and fed twice a week through a silicone membrane with fresh sheep blood in heparin as previously described (Rotureau et al., 2011).

Flies were starved for at least 24 h before being dissected at different time points from 1 to 14 days after ingestion of the infected meal. The head of each fly was first dissected and the proboscis placed into a drop of phosphate buffered saline (PBS) in order to separate the labium, labrum, and hypopharynx (Lloyd and Johnson, 1924). The base of the thecal bulb and/or the cibarium were then dissected in a distinct drop and/or directly examined under the microscope. Whole tsetse alimentary tracts, from the distal part of the foregut to the malpighian tubules, were finally dissected and arranged lengthways in a third PBS drop. Parasites recovered from these organs were treated for further experiments no more than 15 min after dissection. In all figures but Figure 1C, parasite origin is indicated with the following codes: posterior and anterior midguts are referred to as Midgut; proventriculus and foregut are referred to as Foregut; cibarium and base of thecal bulb are referred to as Cibarium; labrum, labium, and hypopharynx are referred to as Proboscis.

Spit samples were obtained from group of 10 flies essentially as described by Peacock et al. (2012). Flies were starved for at least 48 h before being allowed to probe onto an alcohol-cleaned microscope slide on a heating plate held at about 37°C. Saliva samples dried immediately on contact with the microscope slide and slides were stored in the dark at ambient temperature before examination. The samples were checked for the presence of trypanosomes under phase contrast (40x magnification) and positive slides were treated for IFA as described hereafter.

For transmission experiments, flies fed on mice infected with *T. vivax* 7 days earlier, were allowed to feed on uninfected mice. Uninfected mice were first anesthetized by i.p. injection of 100 μl of a mix solution of Ketamine (Imalgene1000 at 125 mg per kg bodyweight) and Xylazine (Rompun 2% at 12.5 mg per kg bodyweight) and individually laid for 20 min on the upper net of a Roubaud cage containing no more than 15 tsetse flies. Parasitaemia was subsequently monitored daily over 1 month. Two replicates were performed with groups of 10 mice each.

### Immunofluorescence

One limiting factor in the present study was the paucity of parasites that could be exploited from biological samples. Therefore, in order to concentrate parasites prior to immunofluorescence analysis (IFA), several protocols were tested: infected proboscis were first pooled and (i) vortexed at low speed for 5 s and/or treated with proteinase K (Sigma) to favor parasite detachment, (ii) and/or collected using Cytospin centrifuge cartridges (Thermofisher) to concentrate cells in smaller areas of the slides. Nevertheless, none of these attempts provided convincing improvements in terms of intact parasite yields (data not shown).

For immunofluorescence, cells were treated as previously described (Rotureau et al., 2011). Parasites were settled on poly-L-lysine coated slides and fixed in 4% para-formaldehyde (PFA) for 10 min. Fixed cells were permeabilised with 0.5% Nonidet P-40 in PBS for 10 min and samples were rinsed to remove the excess of detergent. Blocking was performed by an incubation of 45 min in PBS containing 1% bovine serum albumin. Alternatively, cells were fixed in methanol at −20°C for 10 s and re-hydrated in PBS for 10 min. In all cases, slides were incubated with primary antibodies diluted in PBS containing 0.1% bovine serum albumin for 45 min at 37°C. They were washed and incubated with the appropriate secondary antibodies coupled to Alexa 488 (Invitrogen) or Cy3 (Jackson). Slides were stained with 4″,6-diamidino-2-phenylindole (DAPI) for visualization of kinetoplast and nuclear DNA content, and mounted under cover slips with ProLong antifade reagent (Invitrogen).

Mab25 (mouse IgG2a, no dilution) labels a protein found all along the *T. b. brucei* axoneme (Pradel et al., 2006), while the monoclonal antibody L8C4 (mouse IgG1, no dilution) specifically recognizes *T. b. brucei* PFR2, localized throughout the PFR (Kohl et al., 1999). Anti-TS2 and anti-TS3 antibodies (rabbit polyclonal, 1/200) targeting *T. vivax* surface trans-sialidases (Ammar et al., 2013), calflagin antiserum (mouse, 1/500) that labels all proteins of the calflagin family (Giroud et al., 2009), and two VSG antisera (mouse, 1/500) raised against *T. vivax* variant surface glycoproteins (SG, unpublished material) were used to identify metacyclic and bloodstream trypomastigote parasites. For each antibody, IFA experiments were repeated on trypanosomes issued from at least 3 different experiments.

### Measurements and normalization

Samples were observed either with a DMR microscope (Leica) and images were captured with a CoolSnap HQ camera (Roper Scientific), or with a DMI4000B microscope (Leica) and images were acquired with an ORCA-03G camera (Hamamatsu). Image acquisition was controlled using Micro-manager and images were taken with the min/max threshold set at maximum. Subsequent normalization of signals was carried out by parallel manipulation of min/max signal against controls in ImageJ (NIH), and images were superimposed using Photoshop CS5. For clarity purposes, brightness and contrast of several pictures presented in figures were adjusted after their analysis in accordance with editorial policies. As previously described (Rotureau et al., 2011), all biometric measurements were done with ImageJ V1.49h (http://imagej.nih.gov/ij/): total cell length (T), flagellum length (F), nucleus center to posterior end (N-Post) and nucleus center to kinetoplast (N-K), inter-nuclei distance (N1-N2), and inter-kinetoplasts distance (K1-K2). To homogenize analyses and identify cell morphotypes in a blind manner, nuclei, kinetoplasts, and flagella were numbered along the antero-posterior axis independently from any other considerations. The scale bars represent 10 μm in all figures. Lengths and distances are given as means ± standard deviation (SD) in microns (μm).

### Statistical analyses

**Figure 2:**
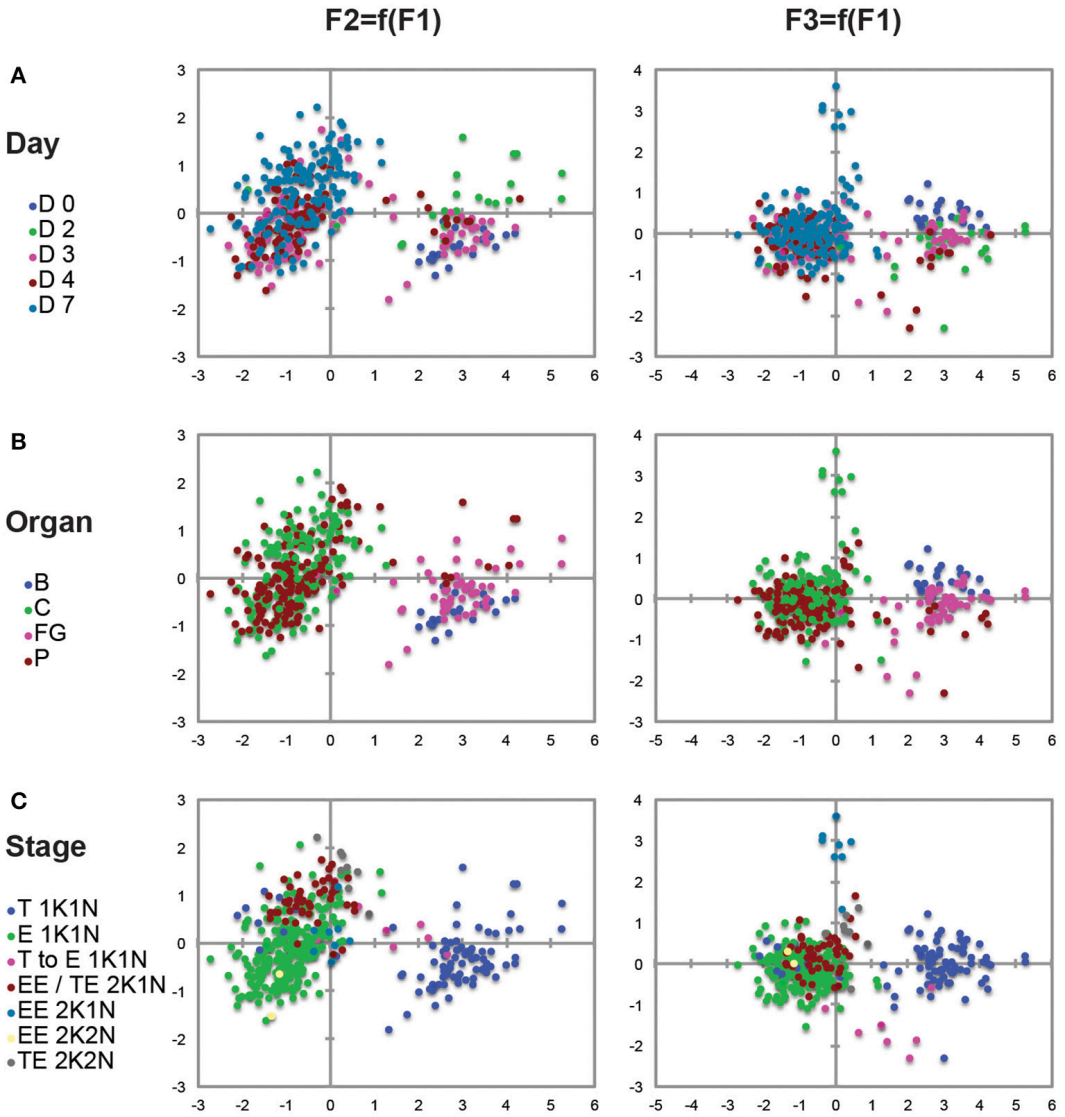
**Principal components analysis (PCA)**. Each plot shows scores for PCA factor 1 vs. factor 2 (left raw) and factor 1 vs. factor 3 (right raw) defined in Figure S1 and derived from the 1594 biometric data measured on 407 individual trypanosomes representative for each time point and organ (detailed measurements in **Tables 2**, **3**; Tables S1, S2). Factor 1 alone accounted for 72.3% of the observed variance and the total cell length (30.3%) and the N-Post distance (24.7%) were the most discriminant biometric parameters. Each trypanosome is represented by a colored dot according to the day after ingestion of the infected bloodmeal **(A)**, the organ **(B)**, or the stage **(C)**. A significant number of 2K1N cells were included in a distinct EE/ET group because they were presenting one kinetoplast in the epimastigote configuration whereas the lateral and perinuclear localization of the second one did not allow a clear resolution between the Epi and Trypo configuration. To homogenize analyses and identify cell morphotypes in a blind manner, nuclei, kinetoplasts, and flagella were numbered along the antero-posterior axis independently from any other considerations. B, blood; FG, foregut; C, cibarium; P, proboscis; T, trypomastigote; E, epimastigote; EE, Epi-Epi dividing cell; TE, Trypo-Epi dividing cell; K, kinetoplast; N, nucleus.

Statistical analyses and plots were performed with Microsoft Excel 2011 and with the XLSTAT 2015.4.01 sofware (Addinsoft). Since many of the dimensions measured were likely to be internally correlated to some extent, all measured variables were preliminary checked for normal distribution and the entire measurement data-set was log-transformed for range homogenization in order to be subjected to principal components analysis using the XLSTAT 2015.4.01 sofware (Addinsoft). To extract underlying latent variables, a Pearson ACP was performed with three factors. Loadings were extracted and the absolute values plotted to determine the extent to which each of the individual measurements contributed to the three factors (Figure S1B). Factor 1 alone was accounting for 72.3% of the observed variance with an eigenvalue of 2.894 and in which the total cell length (30.3%) and the N-Post distance (24.7) were the most discriminant biometric parameters (Figure S1A). Then, extracted scores for all three factors were plotted for each trypanosome (Figures 2A–C). Each plot shows scores for PCA factors compared two by two and derived from the 1594 biometric measurements from 407 individual trypanosomes, each represented by a single dot. Dissection time points (Days 0, 2, 3, 4, and 7 after the infective meal in Figure 2A), parasite origins (organs and tissues in Figure 2B: Blood, Foregut, Cibarium, and Proboscis) and parasite stages (morphotypes and cell cycle steps in Figure 2C) were included as supplemental variables and represented by the indicated color code for each parasite (Figures 2A–C).

## Results

### Infection rates and parasites densities

In order to investigate how *T. vivax* trypanosomes proliferate and differentiate in the cibarium and proboscis, a total of 3650 *Glossina morsitans morsitans* teneral males were fed on Swiss mice infected with *T. vivax* IL 1392 in batches of 35–57 flies across 31 experiments.

To characterize our experimental model, tsetse infection rates were first checked under the microscope over the course of infection by serial dissections of the midgut, foregut, cibarium, and proboscis over 2 weeks after the infective meal (Figures 1A,B). One day after the infective meal, limited numbers of parasites were observed in almost all flies and in all organs but the hypopharynx (Figure 1C). Infection rates in the midgut and foregut then decreased gradually and concomitantly to the bloodmeal digestion process and were undetectable after 4 days. During the same period, infection rates in the cibarium and proboscis ranged between 11 and 52% of dissected flies. Comparable infection rates in these two organs were still observed up to 21 days after infection. Parasites were observed in the hypopharynx at days 3 and 4 after the infective meal in only 2% of the flies (Figure 1C).

Parasites of the cibarium and proboscis were mostly seen attached with their flagellum to the chitinous lining. Sparsely distributed along the entire length of the proboscis up to 2 days after ingestion, they were then seen grouped in one or two small islets restricted to the proximal part of the proboscis during the next few days (Figure 1B, Movies S1, S2). Indeed, a strong feature of the infection was the low parasite density in all organs of infected flies and at any time point. The exact number of parasites per fly was therefore determined in the foregut, cibarium, and proboscis of 3 groups of flies 3, 4, and 7 days after parasite ingestion. To allow for proper cell counting in trypanosome clusters, parasites were first flushed from dissected organs and all the extruded cells as well as those that were still attached to these organs were counted (Figure 1D). The maximum total number of parasites counted in a single fly was only 120 (day 7). From days 3 to 7 after ingestion, parasites progressively disappeared from the foregut, whereas their number increased up to 88 in the cibarium and 72 in the proboscis of a single fly.

### Parasite stages and populations

**Figure 3:**
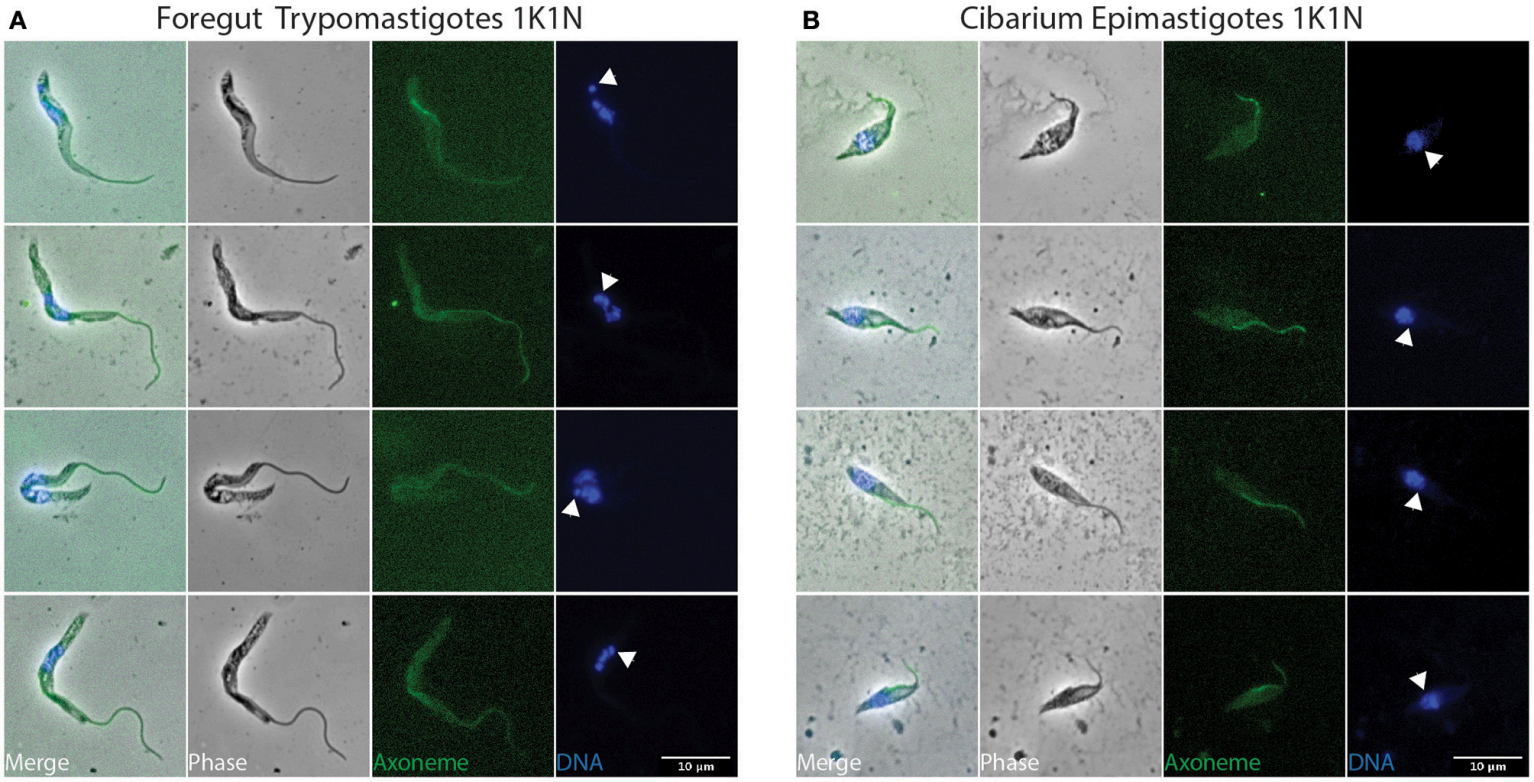
**Early stages in the foregut and cibarium**. *T. vivax* parasites extracted from the foregut and cibarium of infected flies before 3 days post-ingestion of the infected bloodmeal were fixed in methanol and stained with DAPI (DNA in blue) and the Mab25 antibody (axoneme in green). The scale bars represent 10 μm and arrows indicate the kinetoplasts. Only non-dividing (1K1N) trypomastigotes were seen in the foregut **(A)** and non-dividing (1K1N) epimastigotes in the cibarium **(B)**. K, kinetoplast; N: nucleus.

**Figure 4:**
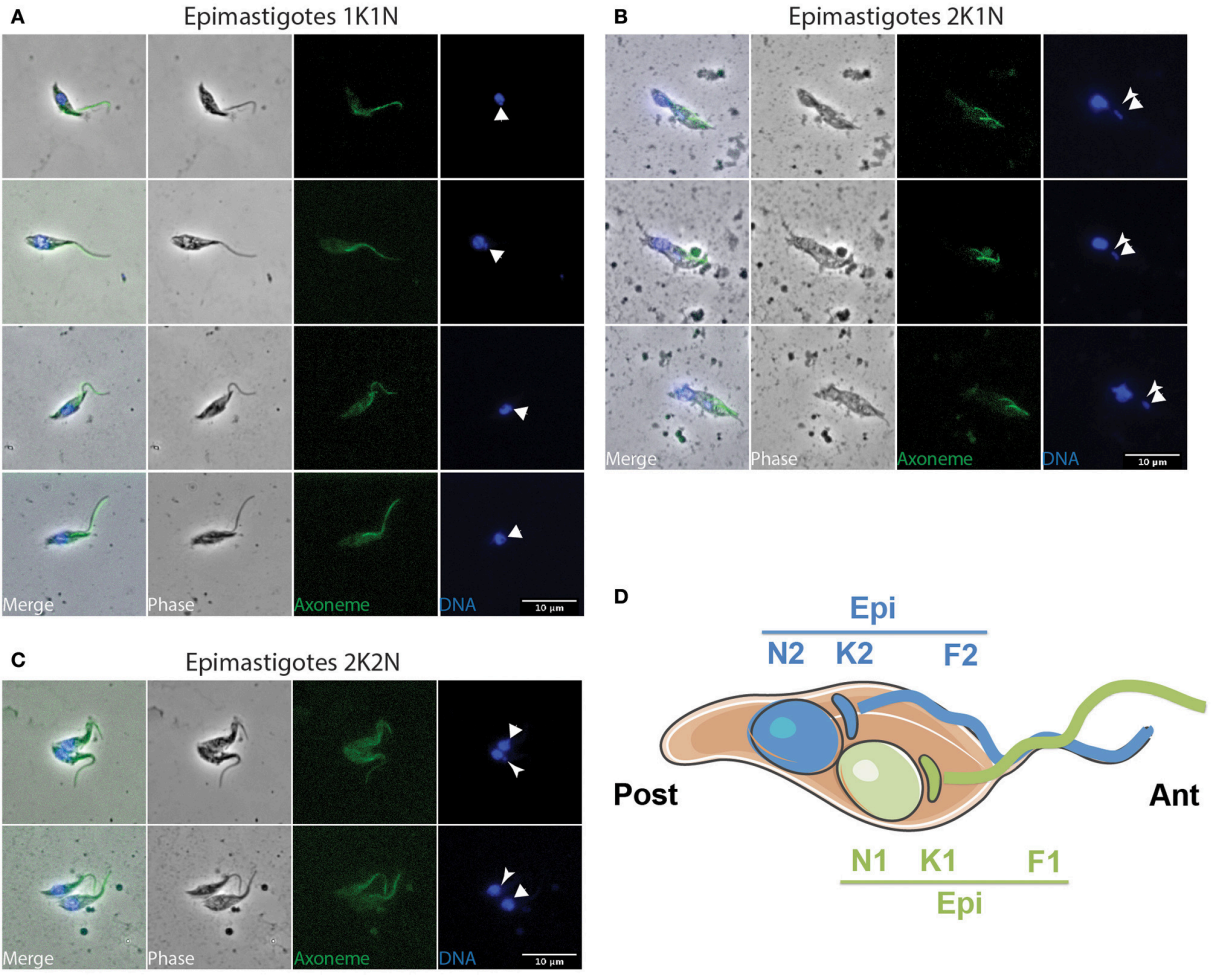
**Epi-epi dividing parasites in the cibarium and proboscis**. *T. vivax* parasites extracted from the cibarium and proboscis of infected flies 4–7 days post-ingestion of the infected bloodmeal were fixed in methanol and stained with DAPI (DNA in blue) and the Mab25 antibody (axoneme in green). The scale bars represent 10 μm and the old/anterior (arrow) and new/posterior (arrowhead) kinetoplasts are indicated. Cells are presented according to their situation in the cell cycle: **(A)** 1K1N, **(B)** 2K1N, and **(C)** 2K2N. **(D)** Cartoon showing the organization of a 2K2N Epi-Epi cell dividing symmetrically. To homogenize analyses and identify cell morphotypes in a blind manner, nuclei, kinetoplasts, and flagella were numbered along the antero-posterior axis independently from any other considerations. E, epimastigote; EE, Epi-Epi dividing cell; K, kinetoplast; N, nucleus; F, flagellum.

**Figure 5:**
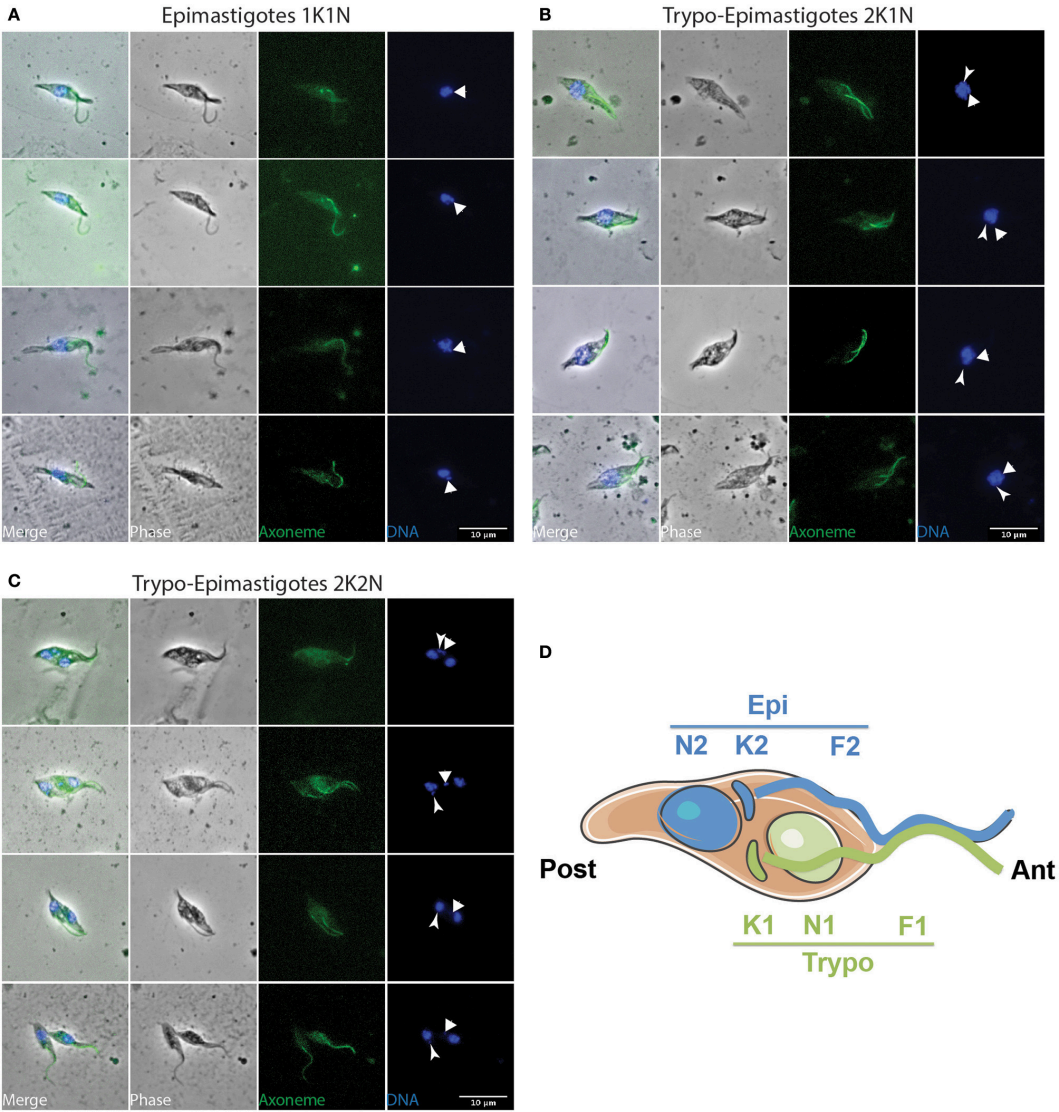
**Trypo-epi dividing parasites in the cibarium and proboscis**. *T. vivax* parasites extracted from the cibarium and proboscis of infected flies 4–7 days post-ingestion of the infected bloodmeal were fixed in methanol and stained with DAPI (DNA in blue) and the Mab25 antibody (axoneme in green). The scale bars represent 10 μm and the old/anterior (arrow) and new/posterior (arrowhead) kinetoplasts are indicated. Cells are presented according to their situation in the cell cycle: **(A)** 1K1N, **(B)** 2K1N, and **(C)** 2K2N. **(D)** Cartoon showing the organization of a 2K2N Trypo-Epi cell dividing asymmetrically. To homogenize analyses and identify cell morphotypes in a blind manner, nuclei, kinetoplasts, and flagella were numbered along the antero-posterior axis independently from any other considerations. T, trypomastigote; E, epimastigote; TE, Trypo-Epi dividing cell; K, kinetoplast; N, nucleus; F, flagellum.

**Figure 6:**
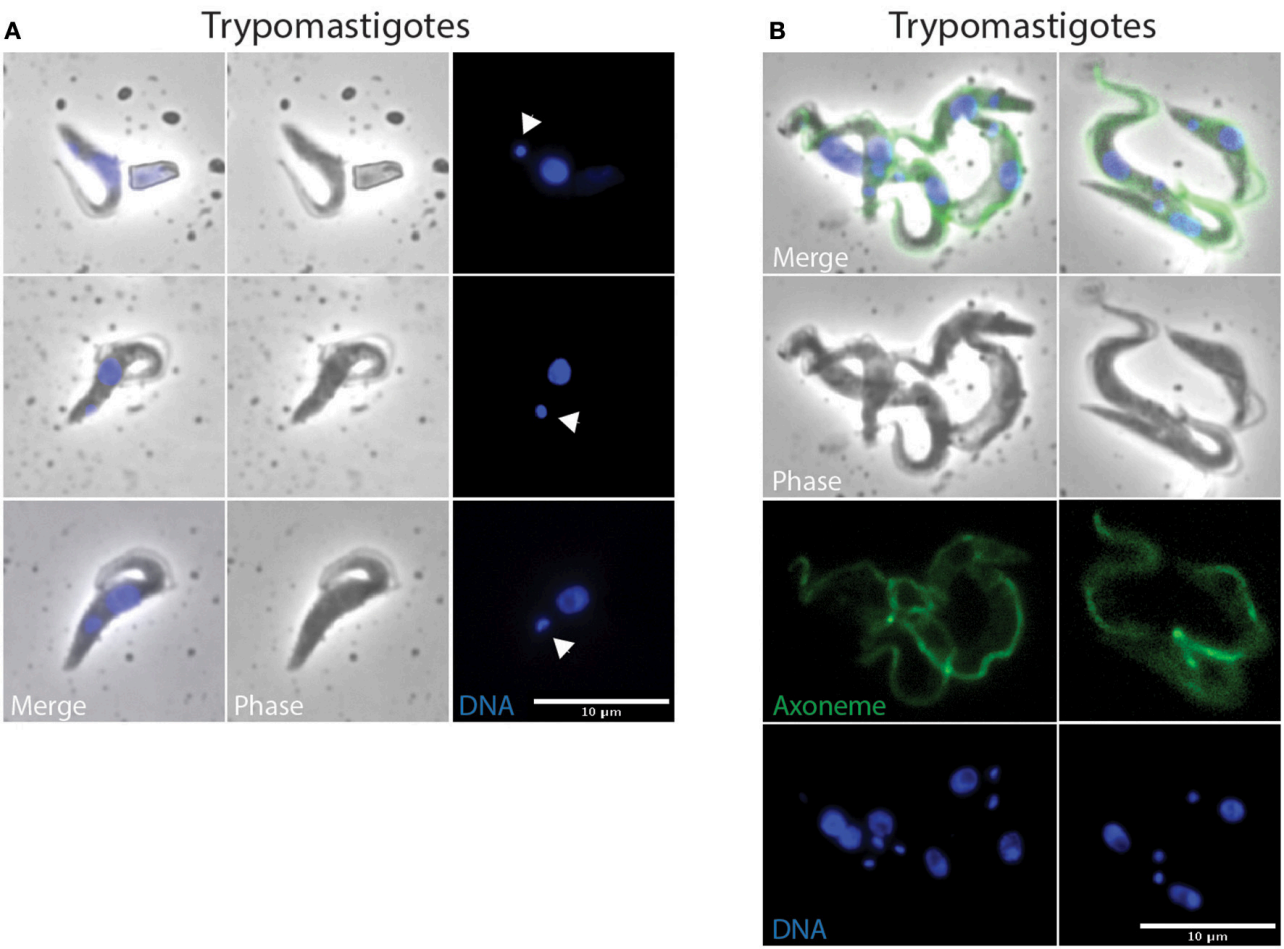
**Metacyclic-like trypomastigote parasites from proboscis and saliva**. *T. vivax* parasites extracted from the proboscis of infected flies 7 days post-ingestion of the infected bloodmeal **(A)** or collected from saliva probes 14 days post-ingestion of the infected bloodmeal **(B)** were fixed in PFA and stained with DAPI (DNA in blue) and the Mab25 antibody (axoneme in green in **B**). The scale bars represent 10 μm and arrows in **(A)** indicate kinetoplasts.

To investigate parasite proliferation and differentiation, infected flies were dissected at different time points after the infective meal (days 2, 3, 4, and 7) and parasites were flushed from tissue (Midgut, Foregut, Cibarium, and Proboscis). Samples were subsequently treated to label their flagellum axoneme (Mab25) and their DNA content (DAPI) (Rotureau et al., 2011). First, the progression of trypanosomes in the cell cycle can be monitored by this simple DNA staining as cells with one kinetoplast and one nucleus (1K1N) are in the G1/S phase, those with two kinetoplasts and one nucleus (2K1N) are in G2/M, and individuals with two kinetoplasts and two nuclei (2K2N) are about to undergo cytokinesis (Sherwin and Gull, 1989; Woodward and Gull, 1990). Second, the use of both DNA (DAPI) and flagellum (Mab25) markers allows for unambiguous distinction between the epimastigote and trypomastigote morphotypes. Therefore, all parasites were carefully examined by microscopy (100x objective) to determine their morphotype and progression in the cell cycle. To identify cell morphotypes in a blind manner, nuclei, kinetoplasts, and flagella were numbered along the antero-posterior axis. Parasite populations were calculated from 1040 cells: representative ones are shown in Figures 3–**6** and Figures S2, S3, and a detailed quantification is provided in Table 1.

**Table 1:** **Parasite populations by stage according to the day after ingestion and the organs**.

		**E 1K1N**			**T 1K1N**			**EE/TE 2K1N**			**EE 2K1N**			**EE 2K2N**			**TE 2K1N**			**TE 2K2N**			**Monsters nFnKnN**			**Total**	
		***n***	**% day**	**% organ**	***n***	**% day**	**% organ**	***n***	**% day**	**% organ**	***n***	**% day**	**% organ**	***n***	**% day**	**% organ**	***n***	**% day**	**% organ**	***n***	**% day**	**% organ**	***n***	**% day**	**% organ**	***n***	***n***
Day 2	FG	–	–	–	23	59%	96%	1	3%	4%	–	–	–	–	–	–	–	–	–	–	–	–	–	–	–	24	39
	C	–	–	–	1	3%	100%	–	–	–	–	–	–	–	–	–	–	–	–	–	–	–	–	–	–	1	
	P	–	–	–	14	36%	100%	–	–	–	–	–	–	–	–	–	–	–	–	–	–	–	–	–	–	14	
Day 3	FG	1	1%	2%	45	29%	96%	–	–	–	–	–	–	–	–	–	–	–	–	–	–	–	1	1%	2%	47	156
	C	47	30%	82%	–	–	–	4	3%	7%	–	–	–	1	1%	2%	3	2%	5%	2	1%	4%	–	–	–	57	
	P	38	24%	73%	5	3%	10%	3	2%	6%	–	–	–	–	–	–	3	2%	6%	3	2%	6%	–	–	–	52	
Day 4	FG	–	–	–	13	4%	93%	–	–	–	–	–	–	–	–	–	–	–	–	–	–	–	1	0%	7%	14	303
	C	71	23%	74%	2	1%	2%	13	4%	14%	–	–	–	–	–	–	9	3%	9%	–	–	–	1	0%	1%	96	
	P	146	48%	76%	4	1%	2%	19	6%	10%	–	–	–	1	0%	1%	19	6%	10%	4	1%	2%	–	–	–	193	
Day 7	FG	–	–	–	–	–	–	–	–	–	–	–	–	–	–	–	–	–	–	–	–	–	–	–	–	–	542
	C	198	37%	69%	7	1%	2%	51	9%	18%	13	2%	5%	–	–	–	10	2%	4%	5	1%	2%	1	0%	0%	285	
	P	186	34%	72%	8	1%	3%	35	6%	14%	10	2%	4%	–	–	–	8	1%	3%	8	1%	3%	2	0%	1%	257	
TOTAL		687			122			126			23			2			52			22			6				1040

*A total of 1040 parasites flushed from 17 infected flies were fixed in methanol and stained with DAPI and the MAb25 antibody for stage determination and counting. For each stage, parasite populations are detailed by parasite counts, proportion of all parasites observed the same day, and proportion of all parasites observed in the same organ, according to the day after ingestion of the infected bloodmeal and to the organ. A significant number of 2K1N cells were included in a distinct EE/ET group because they were presenting one kinetoplast in the epimastigote configuration whereas the lateral and perinuclear localization of the second one did not allow a clear resolution between the Epi and Trypo configuration. To homogenize analyses and identify cell morphotypes in a blind manner, nuclei, kinetoplasts, and flagella were numbered along the antero-posterior axis independently from any other considerations. FG, foregut; C, cibarium; P, proboscis; T, trypomastigote; E, epimastigote; EE, Epi-Epi dividing cell; TE, Trypo-Epi dividing cell; K, kinetoplast; N, nucleus; F, flagellum*.

To allow a better understanding of morphotype transitions, as well as to characterize each distinct stage, 1594 biometric measurements were performed on 407 individual trypanosomes representative for each time point and organ, as previously described (Rotureau et al., 2012). Data for 1K1N cells are shown in Table 2 and Table S1, whereas data for dividing cells are presented in Table 3 and Table S2. In order to get a comprehensive overview of the data set, a principal component analysis (PCA) was performed and presented in Figure 2 and Figure S1. PCA identified the total cell length and the distance between the nucleus and the posterior end as the most determining biological parameters to characterize parasite stages (Figure S1A).

**Table 2:** **Morphometric measurements in non-dividing 1K1N stages**.

**Stage**	**Total**			**F1**			**K1-N1**			**N1-Post**		
	**Mean**	***SD***	***n***	**Mean**	***SD***	***n***	**Mean**	***SD***	***n***	**Mean**	***SD***	***n***
BSF T 1K1N	17.5	2.9	23	21.0	2.6	23	5.0	0.7	22	6.3	1.1	23
Foregut T 1K1N	19.3	5.0	64	21.5	3.3	64	3.3	1.2	64	7.3	2.0	64
E 1K1N	10.1	1.5	224	10.5	1.5	224	1.4	0.3	224	3.6	0.9	224
Proboscis T 1K1N	9.9	2.5	10	8.6	2.6	10	1.2	0.3	10	4.3	0.8	10

*A total of 321 parasites representative of the 4 identified non-dividing stages were fixed in methanol and stained with DAPI and the Mab25 antibody for biometric measurements. This table summarizes all the morphometric measurements performed in the present study in 1K1N parasites. The mean lengths ±SD and numbers of cells studied are given in μm for 4 parameters: the total length of the cell from the tip of the flagellum to the posterior end of the cell (Total), the length of the flagellum (F1), the distance between the center of the nucleus and the kinetoplast (K1-N1), the distance between the center of the nucleus and the posterior end of cell (N1-Post). BSF, bloodstream forms; T, trypomastigote; E, epimastigote; K, kinetoplast; N, nucleus; F, flagellum*.

**Table 3:** **Morphometric measurements in dividing stages**.

**Stage**	**Total**			**F1**			**K1-N1**			**N1-Post**			**F2**		
	**Mean**	***SD***	***n***	**Mean**	***SD***	***n***	**Mean**	***SD***	***n***	**Mean**	***SD***	***n***	**Mean**	***SD***	***n***
EE/TE 2K1N	12.1	1.3	46	8.2	1.3	46	1.4	0.4	46	4.8	0.8	46	6.1	2.7	46
EE 2K1N	14.4	1.3	23	5.5	0.6	8	6.3	1.6	23	3.3	1.0	23	7.3	2.4	8
EE 2K2N	9.7	0.8	2	11.2	0.9	2	1.5	0.2	4	3.7	1.5	4			
TE 2K2N	11.9	1.3	10	7.8	1.9	8	2.1	0.5	10	7.6	0.7	10	9.2	1.4	8
**Stage**	**K2-N1**			**K1-K2**			**K2-N2**			**N2-Post**			**N1-N2**		
	**Mean**	***SD***	***n***	**Mean**	***SD***	***n***	**Mean**	***SD***	***n***	**Mean**	***SD***	***n***	**Mean**	***SD***	***n***
EE/TE 2K1N	1.3	0.3	46	1.2	0.6	46									
EE 2K1N	4.4	1.2	23	2.0	1.1	23									
EE 2K2N													2.1	0.1	4
TE 2K2N				2.5	0.6	10	1.3	0.3	10	2.9	0.6	10	4.9	1.1	10

*A total of 83 parasites representative of the 2 types of cell division identified in the cibarium and proboscis were fixed in methanol and stained with DAPI and the Mab25 antibody for biometric measurements. This table summarizes all the morphometric measurements performed in the present study in 2K1N and 2K2N parasites. The mean lengths ±SD and numbers of cells studied are given in μm for 10 parameters (cf. organization of dividing cells in Figures 4D, 5D): the total length of the cell from the tip of the flagellum to the posterior end of the cell (Total), the length of the flagellum associated to the anterior/old kinetoplast (F1), the distance between the center of the anterior nucleus and the anterior/old kinetoplast (K1-N1), the distance between the center of the anterior nucleus and the posterior end of cell (N1-Post), the length of the flagellum associated to the posterior/new kinetoplast (F2), the distance between the center of the anterior nucleus and the posterior/new kinetoplast (K2-N1), the distance between the two kinetoplasts (K1-K2), the distance between the center of the posterior nucleus and the posterior/new kinetoplast (K2-N2), the distance between the center of the posterior nucleus and the posterior end of cell (N2-Post), the distance between the two nuclei (N1-N2). A significant number of 2K1N cells were included in a distinct EE/ET group because they were presenting one kinetoplast in the epimastigote configuration whereas the lateral and perinuclear localization of the second one did not allow a clear resolution between the Epi and Trypo configuration. To homogenize analyses and identify cell morphotypes in a blind manner, nuclei, kinetoplasts, and flagella were numbered along the antero-posterior axis independently from any other considerations. T, trypomastigote; E, epimastigote; EE, Epi-Epi dividing cell; TE, Trypo-Epi dividing cell; K, kinetoplast; N, nucleus; F, flagellum*.

Combination of all these data in space and time allowed us to unravel, at least partially, *T. vivax* cyclical development in the tsetse fly. For clarity purposes, the main observations are detailed hereafter following the bio-chronological progression of parasite development.

### Parasites from the midgut and foregut

Whereas dividing trypomastigote bloodstream forms were easily detected in mouse blood (Figure S2), only non-dividing (1K1N1F) cells were observed in flies during the first 48 h after ingestion (Figure 3A and Table 1). These non-dividing cells were mostly seen in the midgut and especially in the foregut (59%) where they elongated from 17.5 ± 2.9 to 26.4 ± 4.6 μm (Table 2 and Table S1). In apparent transition to the epimastigote stage, nuclear DNA was usually fragmented (Figure 3A). These cells in the midgut and foregut decreased by days 3 and 4 post-infection (Table 1).

### Parasite from the cibarium and proboscis

Non-dividing (1K1N1F) epimastigotes were observed from day 3 after the infective meal in the cibarium and proboscis (Figure 3B), accounting for 30 and 24% of the parasite population respectively (Table 1). They were detected in increasing numbers in these organs up to day 7 (Table 1).

Concomitantly, dividing epimastigotes were detected from day 3 after the infective meal (Table 1 and Figure 4). A significant number of 2K1N cells were presenting one kinetoplast in the epimastigote configuration whereas the lateral and perinuclear localization of the second one did not allow a clear resolution between the epimastigote and trypomastigote configuration (126 EE/TE in Table 1). In total, 2K1N2F epimastigotes (Figure 4B) represented up to 23% of the parasites present in the cibarium after 7 days (EE/TE 2K1N + EE 2K1N in Table 1).

From microscopic observations (Figure 4 and Figure S3) and morphometric measurements (Table 3 and Table S2), it appears that *T. vivax* dividing epimastigote cells are similar to that observed in *T. brucei brucei* epimastigotes attached to the salivary glands (Rotureau et al., 2012), with one epimastigote cell producing two similar epimastigote daughters. Nevertheless, only two Epi-Epi 2K2N cells were observed during the experiment (Table 1 and Figure 4C). Compared to the total of 149 2K1N epimastigote cells, this low number of 2K2N epimastigote parasites suggests that mitosis and/or cytokinesis are rapid events in this cell cycle, or that these cells could be more fragile. Another explanation for these unbalanced numbers of 2K1N vs. 2K2N dividing epimastigote cells was provided by a striking observation in neighboring cells that were undergoing another type of division.

Indeed, an equivalent proportion of epimastigote cells (between 1 and 5% of the total per organ) were seen dividing asymmetrically (Figure 5). In these parasites, the posterior kinetoplast associated to the old flagellum (K2 and F2) were in the epimastigote (Epi) position, whereas the anterior kinetoplast and new flagellum (K1 and F1) were in the trypomastigote (Trypo) configuration (Figures 5C,D). These Trypo-Epi cells were observed as early as 3 days after the infective meal and a total of 52 2K1N2F and 22 2K2N2F cells were counted in the cibarium and proboscis of the dissected flies (Table 1).

When comparing the cellular events involved in these two types of division, we observed that, after segregation, kinetoplasts were seen to migrate anteriorly and to reposition along the antero-posterior axis of the cell in Epi-Epi parasites (K1N1 6.3 ± 1.6 μm and K2N1 4.4 ± 1.2 μm at almost constant N1-Post distance). In contrast, the kinetoplasts of Trypo-Epi cells were seen to segregate up to 2.5 ± 0.6 μm in the vicinity and on each side of the nucleus (K1N1 2.1 ± 0.5 μm and K2N1 2.5 ± 0.6 μm at almost constant N1-Post distance) while this nucleus was migrating anteriorly (N1-Post 7.6 ± 0.7 μm). Then, mitosis occured in the antero-posterior axis in Epi-Epi cells where the two nuclei remained very close to each other until cytokinesis (2.1 ± 0.1 μm). These 2K2N Epi-Epi cells elongated up to 14.4 ± 1.3 μm with both parent and daughter flagella of equivalent length (Table 3 and Table S2, Figure 4D). In contrast, mitosis occurs in a more transversal manner in Trypo-Epi cells with one nucleus (N1) migrating anteriorly (N1-N2 4.9 ± 1.1 μm). No cell elongation but a widening was observed in these 2K2N Trypo-Epi cells and their flagella were also of equivalent length (Table 3 and Table S2, Figure 5D).

### Pre-metacyclic forms

If the Epi-Epi division contributes to the colonization and maintenance of the epimastigote population found attached to the cibarium and proboscis, it is tempting to associate the Trypo-Epi division to the production of precursors of infective metacyclic trypomastigotes or pre-metacyclics. In order to verify this hypothesis, a panel of bloodstream form markers were used for immunofluorescence screening: the anti-TS2 and anti-TS3 antibodies targeting *T. vivax* surface trans-sialidases (Ammar et al., 2013), a calflagin antiserum that labels all proteins of the calflagin family in *T. brucei* (Giroud et al., 2009), and two VSG antisera raised against *T. vivax* variant surface glycoproteins (SG, unpublished material). Nevertheless, only the anti-TS3 and anti-VSG1 antibodies were able to label bloodstream forms (Figures S4A,B) or trypomastigotes in culture (Figure S4C) and no significant signal was detected in any cells extracted from the cibarium and proboscis of infected flies.

Limited numbers of 1K1N trypomastigotes were observed in the cibarium and proboscis of infected flies from day 3 after the infective meal (Table 1 and Figure 6A). Over the course of infection, their proportion remained constant around 2 to 3% of the total cells (Table 1). Moreover, among all the flies dissected in this study, only two trypomastigotes were detected in the hypopharynx. We reasoned that this low number could be biased by the possible release of free-swimming metacyclic cells in the PBS drop during dissection and before microscopic observation. To verify this hypothesis, 90 flies were fed on 2 infected mice, maintained for 12 days, starved for 48 h, and finally allowed to probe on warm glass slides 14 days after ingestion of the infective bloodmeal. Saliva drops were first screened under the microscope to check for the presence of metacyclic forms and mouthparts were then dissected to verify the fly infection status (Figure 6B). Although 26% of the flies were found infected, only 10 trypomastigotes, morphologically similar to pre-metacyclic parasites, were detected on slides where flies were allowed to probe, therefore confirming the scarcity of these forms.

In order to test their transmission efficiency, batches of 15 flies were fed on mice infected with *T. vivax* and maintained for 7 days before the transmission experiment. In these conditions, each batch should theoretically contain at least 3 infected flies (around 26%). Flies were allowed to feed on anesthetized uninfected mice for 20 min and parasitaemia was then individually monitored at least 4 times per week over 1 month. Two replicates with groups of 10 mice were performed, nevertheless, no parasite transmission was observed. This result was in accordance with the reduced/absent cyclical transmission of *T. vivax* in mice reported in the literature (De Gee et al., 1976; Leeflang et al., 1976; Moloo, 1981).

### Controls from *in vitro* culture

**Figure 7:**
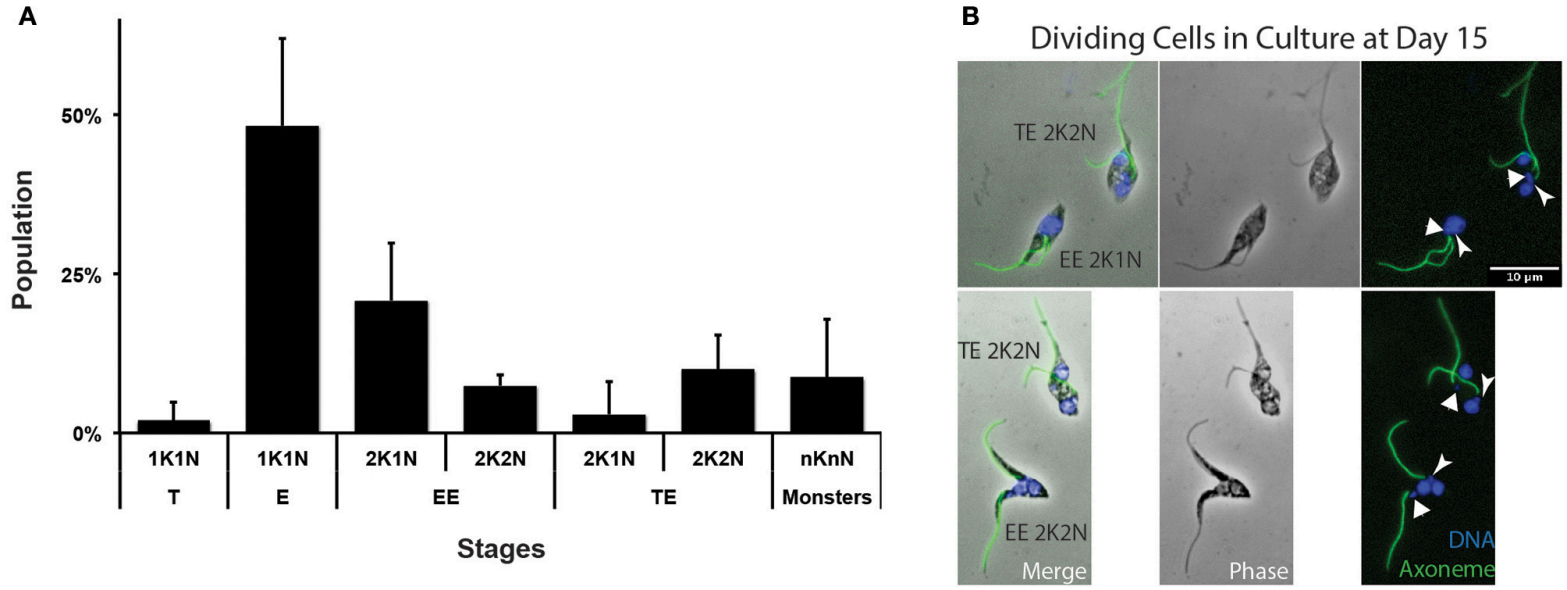
*****T. vivax*** dividing forms in culture**. *T. vivax* parasites were cultured for 15 days, fixed in methanol and stained with DAPI (DNA in blue) and the Mab25 antibody (axoneme in green). **(A)** Parasite populations were plotted by stage and cell cycle step (in % of the total cells, *n* = 293 cells). **(B)** Representative Epi-Epi and Trypo-Epi dividing cells. The scale bars represent 10 μm and the old/anterior (arrow) and new/posterior (arrowhead) kinetoplasts are indicated. T, trypomastigote; E, epimastigote; EE, Epi-Epi dividing cell; TE, Trypo-Epi dividing cell; K, kinetoplast; N, nucleus.

In the absence of molecular markers for metacyclogenesis and without any evidence for efficient transmission from tsetse to mice, we reasoned that another way to confirm the origin of metacyclic forms would be with an *in vitro* approach. To this end, the recent optimization and standardization of non-infective *T. vivax* epimastigote axenic cultures that lead to *in vitro* differentiation into metacyclic infective forms was used (D'Archivio et al., 2011). Axenic *T. vivax* cultures were sampled 15 days after seeding, i.e., at the moment where metacyclic parasites were the most abundant, and treated for IFA to label trypanosome flagella. Populations were quantified according to cell morphotypes and division status (Figure 7A, *n* = 293 cells). Although 1K1N epimastigotes were the most abundant (48%), 1K1N metacyclic trypomastigotes accounted for 2% of the cells, and 2K1N dividing epimastigotes for 24%. Interestingly, 2K2N dividing cells were always seen in one of the two configurations previously observed *in vivo*: 7% were Epi-Epi and 10% were Trypo-Epi dividing trypanonosomes (Figure 7B). A proportion of the parasite population with a juxtanuclear kinetoplast would suggest a gradual migration of the kinetoplast from an epimastigote to a trypomastigote position. Since neither dividing trypomastigotes nor 1K1N trypomastigotes with juxtanuclear kinetoplast were detected, it is unlikely that kinetoplast migration in non-dividing cells produce infective metacyclic trypomastigotes and these cells probably originated mostly from asymmetrical Trypo-Epi divisions.

## Discussion

**Figure 8:**
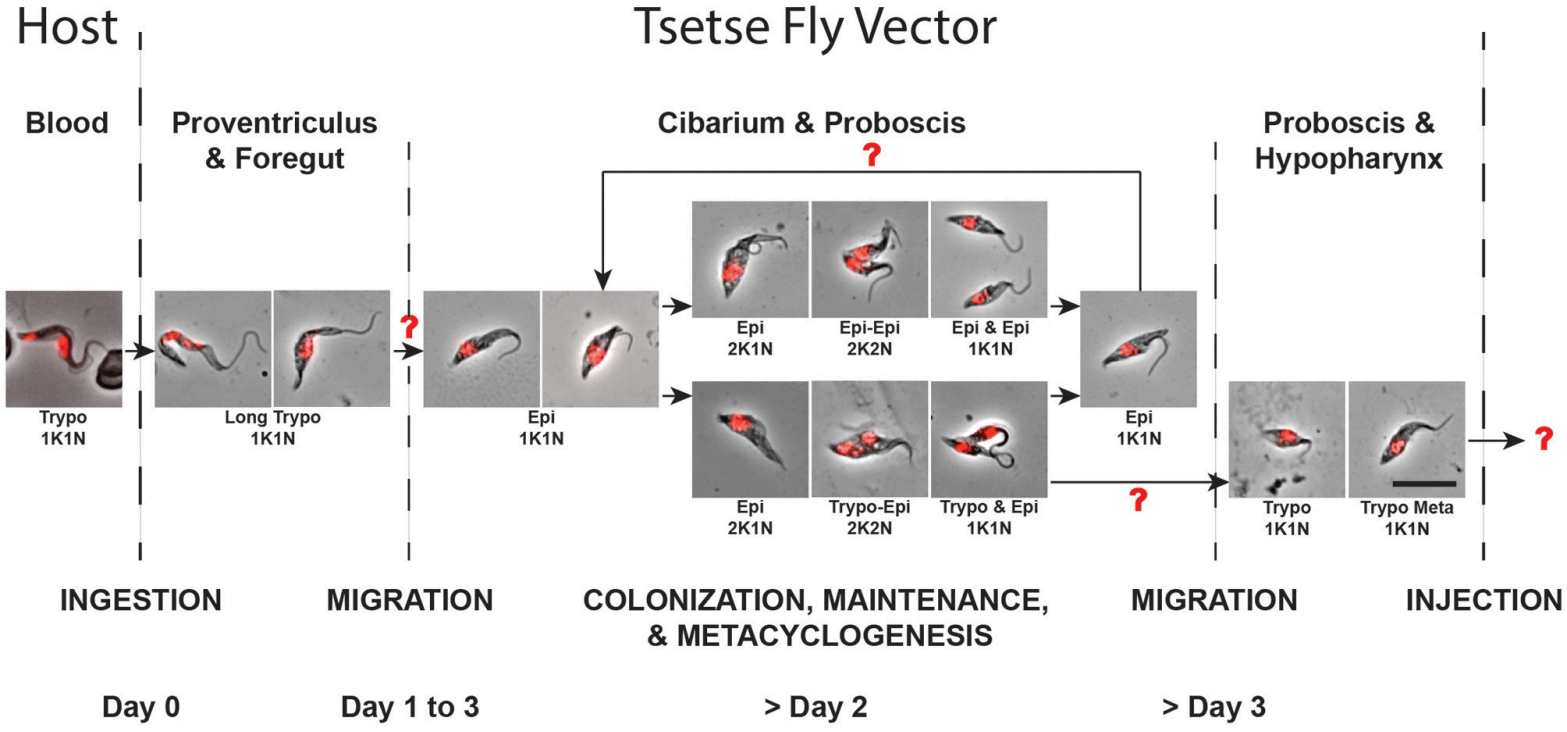
**Proposed model for the ***T. vivax*** cyclical development**. Parasites extracted from infected tsetse flies were fixed in methanol and stained with DAPI (DNA in red). The scheme represents all the observed parasite stages according to the kinetics of the infection and to their localization (by host and organ). Arrows indicate transition between two successive stages and red question marks highlight transitions remaining partially or fully undetermined. The scale bar represents 10 μm. Trypo, trypomastigote; Epi, epimastigote; K, kinetoplast; N, nucleus.

During the last decade, a significant number of reviews have presented in-depth overviews of our knowledge on trypanosome cyclical development and tsetse-trypanosome interactions focusing almost exclusively on *T. brucei* parasites (Aksoy and Rio, 2005; Roditi and Lehane, 2008; Sharma et al., 2009; Walshe et al., 2009; Dyer et al., 2013; Ooi and Bastin, 2013). Nevertheless, these reviews have neglected to address the life cycle of *T. vivax*, which is the dominant species of trypanosome in West Africa in terms of geographic distribution and prevalence, and is the major pathogenic trypanosome of cattle (Gardiner and Wilson, 1987; Osorio et al., 2008; FAO, 2015). This is likely due primarily to a lack of interest in a parasite that is non-pathogenic to humans, but also to major technical issues such as its non-amenability to cell culture and transformation, to the usually low number of parasites present in infected tsetse flies as well as to its poor vector transmissibility to laboratory mice. Using an experimental approach based on large numbers of tsetse infections, we provide new observations on *T. vivax* cyclical development in the tsetse that led us to propose an updated model in Figure 8, assuming that some important parts of this scheme still remain to be clarified or fully determined.

### *T. vivax* development

In *T. vivax*, a single type of trypomastigote is able to proliferate in the bloodstream of the mammalian host. After a tsetse ingests an infected bloodmeal, bloodstream trypomastigotes degenerate in the midgut during digestion within a few days, while only a small number of elongated trypomastigote forms remain in the foregut and cibarial regions. As assessed by their nuclear DNA fragmentation and their progressive disappearance, trypomastigotes remaining in the foregut are certainly doomed to die. There are apparently no proliferative trypomastigote stages as in *T. congolense* and *T. b. brucei*, and our PCA analysis revealed no clear continuum from these early foregut trypomastigotes to the epimastigote population found in the cibarium and proboscis. One unlikely hypothesis is that the first trypomastigote to epimastigote differentiation may occur early (during the first 48 h), in a transient asymmetrically dividing stage, although evidence for this remains elusive (Jefferies et al., 1987; Moloo and Gray, 1989). A second hypothesis would be a rapid differentiation in a 1K1N cell, either directly in the cibarium, or after migration from the midgut/foregut back to the cibarium. Epimastigote parasites from the cibarium are then thought to migrate and to attach with their flagellum to the proboscis (Jefferies et al., 1987; Moloo and Gray, 1989).

Epimastigote parasites subsequently multiply at the foci of attachment to form rosettes (Bruce et al., 1911; Roubaud, 1935; Vickerman, 1973), presumably in order to colonize the mouthparts. Upon formation of these rosettes, trypomastigote pre-metacyclic-like cells resulting from a still undetermined differentiation process can also be seen in the alimentary canal in limited numbers. We propose that this metacyclogenic process, originating from an epimastigote cell, could be the result of a Trypo-Epi asymmetric division producing one trypomastigote precursor of the pre-metacyclic form and one epimastigote daughter. The fate of the latter remains unknown, as it is still the case for other asymmetric divisions in *T. brucei* (Van Den Abbeele et al., 1999; Rotureau et al., 2012). Due to the limited total number of parasites available in tsetse infection and the concomitant occurrence of symmetric divisions in other epimastigotes, it is possible that the epimastigote daughters from the Trypo-Epi asymmetric divisions are doomed to die. Moreover, we could hypothesize that the simultaneous occurrence of these two cell cycle types may represent fine regulation of parasite populations as well as in the continuous production of transmissible infective forms.

When the pre-metacyclic trypomastigotes arising from the asymmetric division become detached, they are thought to swim toward and to invade the hypopharynx where they mature into the short infective trypomastigote metacyclic forms (Lloyd and Johnson, 1924; Gardiner and Wilson, 1987; Jefferies et al., 1987; Moloo and Gray, 1989). Alternatively, or concomitantly, infective trypomastigote metacyclic forms remaining in the alimentary canal could also be expelled by vomiting before ingestion of the bloodmeal, in order to increase the final number of infective parasites transmitted to the next host. This would explain the low number of metacyclic and/or pre-metacyclic forms observed in the hypopharynx.

Nevertheless, the generation/identification of more efficient metacyclic-specific markers will be necessary to unequivocally demonstrate the importance of this asymmetric division for metacyclogenesis in future studies, possibly in another model including a more virulent parasite strain.

### Morphotype switch and asymmetric division

A common feature of the tsetse-transmitted African trypanosome developmental programs is the passage through the epimastigote morphotype. One of the key questions was whether any form equivalent to the two asymmetric dividing stages of *T. brucei* could be found for *T. vivax* (Van Den Abbeele et al., 1999; Sharma et al., 2008; Rotureau et al., 2012). Some evidence suggests such mechanisms in *T. congolense* (Peacock et al., 2012) and we have observed that the final transition from epimastigote to trypomastigote is also likely to rely on an asymmetric process in *T. vivax*. It is noteworthy that, in the same context of metacyclogenesis, the Trypo-Epi asymmetric division described here for *T. vivax* appears to be morphologically inverted compared to the Epi-Trypo division we have previously observed in *T. brucei* (Rotureau et al., 2012). Some other distinct types of asymmetric divisions were also recently depicted as crucial parts of the development of other trypanosomatid parasites. During *Leishmania* promastigote to amastigote intracellular differentiation, most of the promastigote flagellum length is lost by asymmetric division (Wheeler et al., 2015). The *T. cruzi* flagellum is discarded via another asymmetric division following invasion of mammalian host cells and provides early targets for protective CD8(+) T cells (Kurup and Tarleton, 2014). More generally, asymmetric cell division is now considered as a mechanism for cell-type diversification in both prokaryotes and eukaryotes, and is assorted to highly coordinated cell-fate segregation, genome partitioning and cytokinesis (Li, 2013).

Nevertheless, the biological reason for this obligatory morphotype switch remains elusive (Rotureau and Van Den Abbeele, 2013). Is the epimastigote morphotype more adapted to cell fixation via the flagellum compared to the trypomastigote? This flagellum attachment to a solid substrate is a prerequisite for multiple parasite transmission as it provides an efficient way to maintain a pool of progenitor cells that continuously produces infective forms without being expelled with the saliva during tsetse fly feeding. The ability of *T. vivax* to establish and maintain firm anchorage on the ostensibly smooth chitinous lining of the food canal is remarkable as this attachment must remain secure during subsequent feeding of the fly when blood is being pumped through the canal under pressure (Vickerman, 1973). The reason for the localization of trypanosomes in the cibarium in the majority of infections can perhaps be explained by the 3-dimensional shape of the cibarium. The posterior of the dorsal wall curves, creating a recess where trypanosome rosettes in light and moderate infections are most often situated. They are probably also affording some protection from the main force of the blood flow through the cibarium during feeding (Jefferies et al., 1987).

This morphotype switch implies a drastic internal re-organization of the nucleus and kinetoplast that might represent a costly cellular event. Therefore, the occurrence of this switch in all these cyclical developmental programs suggests that it plays an essential role in the parasite life cycle. The highly distinct developmental pathways of the three different trypanosome groups could be the result of a long-term co-evolution between parasites and their vectors that minimizes inter-trypanosome competition within the tsetse fly in order to maximize their respective transmission (Rotureau and Van Den Abbeele, 2013). Since *T. vivax* shows generally higher infection rates in tsetse flies than do *T. congolense* or *T. b. brucei*, it would appear that *T. vivax* is better adapted to development in tsetse and is therefore believed to be the most ancient of these salivarian trypanosomes.

The mouse is apparently not a natural mammalian host in which most of the *T. vivax* strains can develop and proliferate (De Gee et al., 1976; Leeflang et al., 1976; Moloo, 1981), hence the absence of transmission in the present study. Nevertheless, we confirm that this high prevalence in tsetse flies is counter-balanced by the irregular frequency of metacyclic parasite release in the saliva and by very low numbers of metacyclic forms extruded in these rare cases (Bruce et al., 1910, 1911; Lloyd and Johnson, 1924; Roubaud, 1935; Otieno and Darji, 1979). This apparently low transmissibility renders *T. vivax* cyclical development as fascinating as frightening, knowing that high transmission levels persist for centuries. To solve this conundrum, more information is therefore urgently needed on the occurrence and relative epidemiological importance of this cyclical development in field-caught tsetse flies.

## Author contributions

CO, SC, CC, EB, AC, SG, SP, and BR contributed to the experiments. CO, SC, and BR contributed to study design, data analysis and manuscript writing.

## Funding

This work was funded by the Institut Pasteur (PTR-403) and by the French National Agency for Scientific Research (Young Researcher Grant ANR-14-CE14-0019-01). CO and CC were funded by a French Government Investissement d'Avenir programme, Laboratoire d'Excellence “Integrative Biology of Emerging Infectious Diseases” (ANR-10-LABX-62-IBEID). SC was funded by a master fellowship from Institut Pasteur. EB was funded by a doctoral fellowship from French National Ministry for Research and Technology (doctoral school CDV515). AC, SG, SP, and BR were funded by Institut Pasteur.

### Conflict of interest statement

The authors declare that the research was conducted in the absence of any commercial or financial relationships that could be construed as a potential conflict of interest.
