# Supplementary figures and images for: The Cyclical Development of Trypanosoma vivax in the Tsetse Fly Involves an Asymmetric Division
Source: Front Cell Infect Microbiol. 2016 Sep 28;6:115. doi: 10.3389/fcimb.2016.00115 (PMC5039179; doi:10.3389/fcimb.2016.00115)

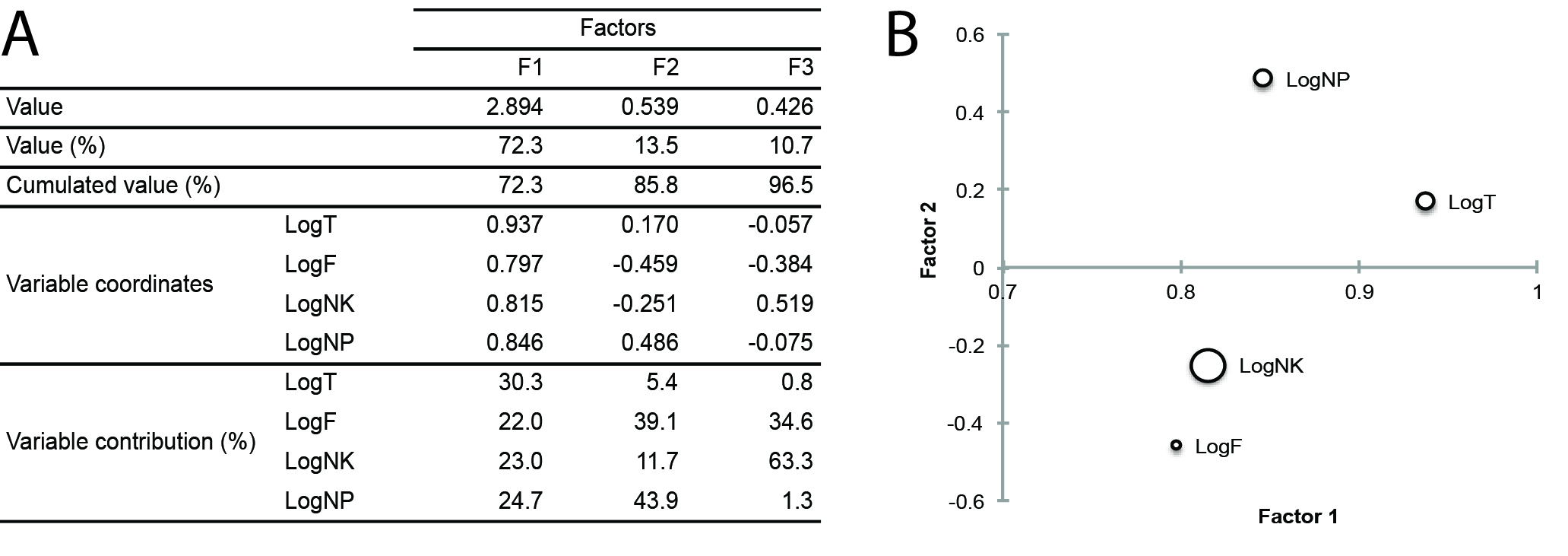

Supplement: Supplementary file 5 [file Image1.JPEG]

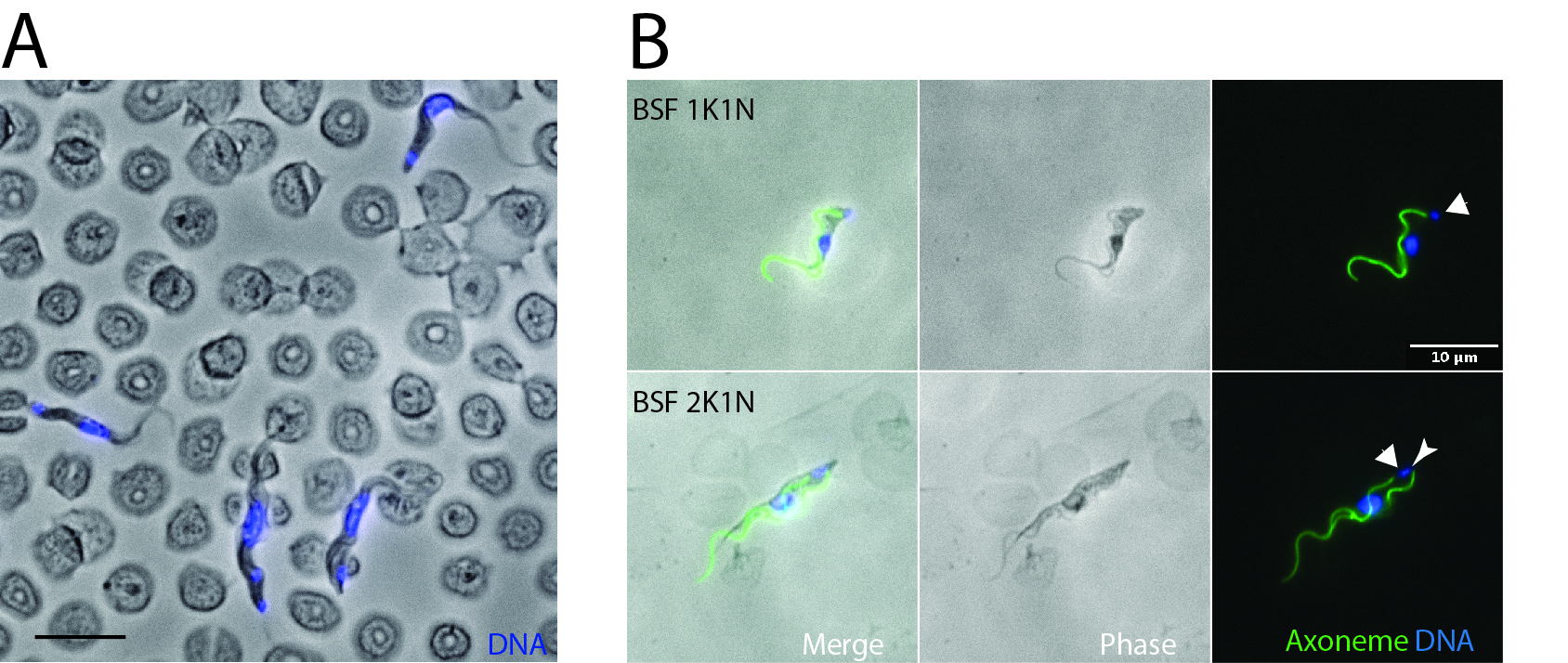

Supplement: Supplementary file 6 [file Image2.JPEG]

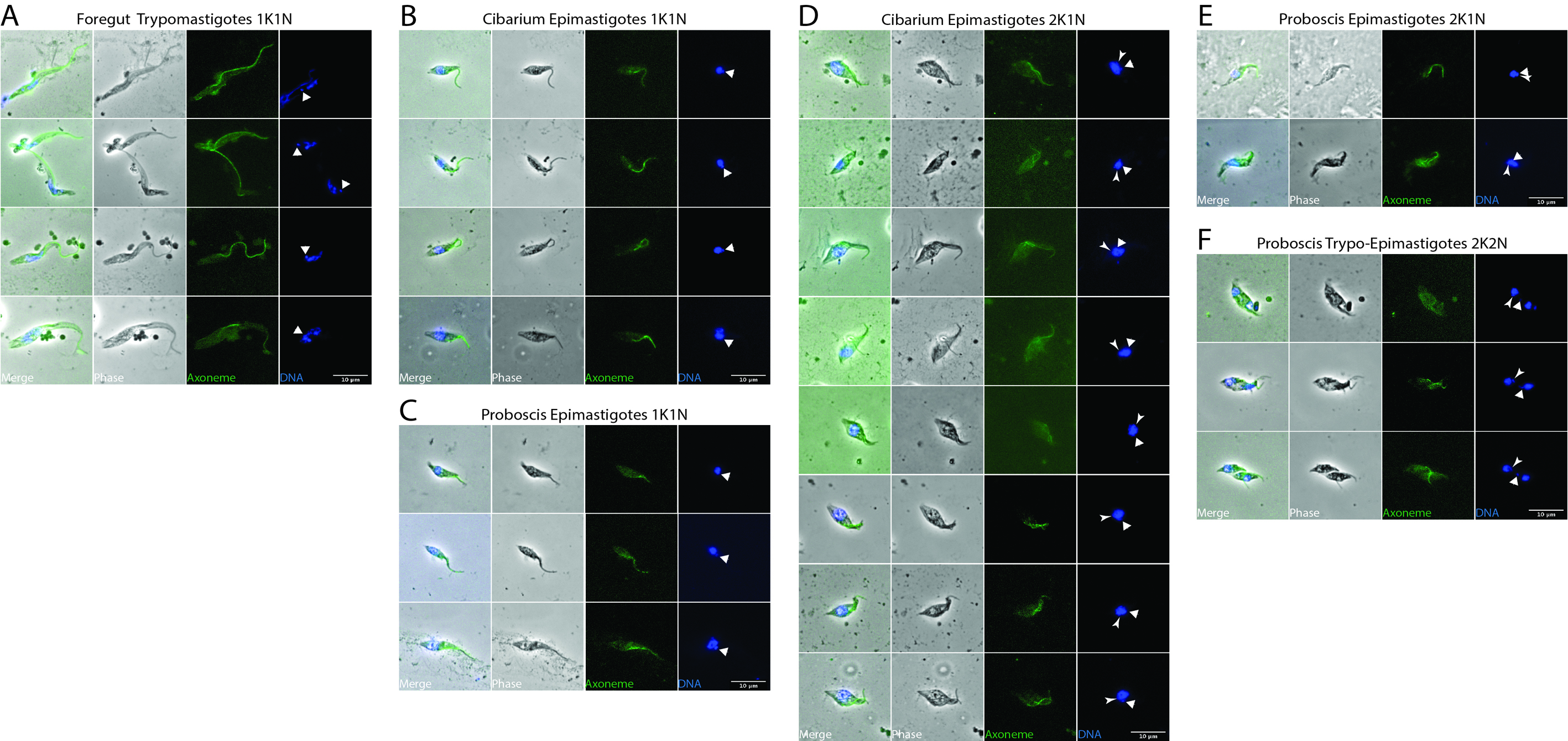

Supplement: Supplementary file 7 [file Image3.JPEG]

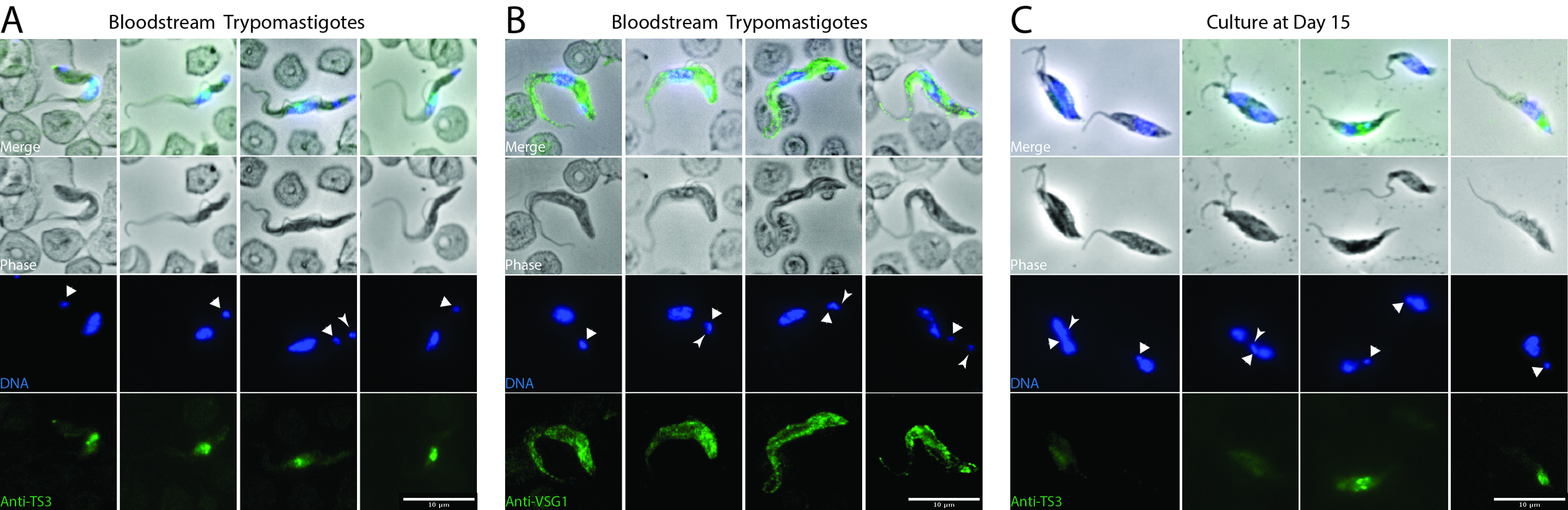

Supplement: Supplementary file 8 [file Image4.JPEG]
